# Habitat explains intraspecific leaf morphological and physiological variation in the facultative epiphyte *Nephrolepis cordifolia*

**DOI:** 10.1007/s10265-026-01737-w

**Published:** 2026-07-20

**Authors:** Rui-Hong Hsu, J. Aaron Hogan, Chiao-Ping Wang, Teng-Chiu Lin

**Affiliations:** 1 Department of Life Science, National, Taiwan Normal University, Taipei, Taiwan; 2 Department of Ecology and Conservation Biology, Texas A&M University, College Station, TX USA; 3 Silviculture Division, Taiwan Forestry Research Institute, Taipei, Taiwan; 4 Department of Natural Resources and Environmental Studies, National Dong Hwa University, Hualien, Taiwan

**Keywords:** Facultative epiphytes, Leaf economics, Leaf functional traits, Water-use efficiency

## Abstract

Epiphytic and terrestrial habitats differ markedly in water, nutrient, and light availability and variability, yet few facultative epiphytes successfully grow in both habitats. As a facultative epiphyte, *Nephrolepis cordifolia* provides a valuable opportunity to investigate how individuals of the same species adjust morphological and physiological traits along contrasting microhabitats. We measured frond structure, physiology, and tissue nutrient concentrations for 20 epiphytic and 20 terrestrial *N. cordifolia* individuals to evaluate intraspecific variation in resource-use strategies by growth habitat. Our findings show that not all Leaf Economic Spectrum traits covary in this species. Structurally, while specific leaf area, leaf dry matter content, and stomatal morphology were conserved across habitats, epiphytic individuals exhibited greater leaf thickness, a trait consistent with conservative leaf economics strategies. Functionally, epiphytes exhibited more enriched *δ*^13^C, indicating higher intrinsic water-use efficiency and tighter stomatal regulation. Terrestrial individuals displayed significantly higher maximum quantum yield of PSII (*F*_v_/*F*_m_) and greater mass-based chlorophyll concentrations, reflecting higher potential photosynthetic capacity. In terms of leaf tissue chemistry, terrestrial individuals had higher nitrogen and magnesium concentrations, while epiphytes showed higher leaf carbon content and significantly elevated C/N ratios. These findings highlight the physiological plasticity of *N. cordifolia*, which enables adjustment to contrasting microhabitats through adjustments in water-use efficiency, photosynthetic capacity, and leaf chemistry. Our results support the idea that epiphytic plants generally exhibit a more conservative physiological profile, with reduced *F*_v_/*F*_m_ and enriched *δ*^13^C, showing how they may function under chronically constrained physiological margins. This vulnerability underscores the potential risks epiphytes face under intensifying drought and irradiance associated with climate change, raising important questions about the resilience of facultative epiphytes in increasingly variable environments.

## Introduction

Environmental conditions strongly influence plant morphology and physiology (Bano et al. 2019; Bohnert et al. 1995; Lambers and Oliveira 2019), such that plants growing in different environments are likely to have different morphological traits and physiological performance. In forests, terrestrial and epiphytic microhabitats exemplify environments, within the same ecosystems, that are very different from one another. Compared to terrestrial habitats, many epiphytic habitats not only have larger fluctuations in irradiance due to less shading by trees but also have more limited water and nutrient availability due to the lack of direct access to soil water and nutrients (Benzing 1990; Watts and Watkins 2021; Zotz and Hietz 2001). These contrasting environmental conditions likely promote different strategies of resource acquisition, carbon economy, and stress tolerance between terrestrial and epiphytic plants, which are likely to be reflected in functional morphology and physiology.

Differences in light and CO_2_ acquisition strategies in vascular plants are well summarized by the leaf economics spectrum (LES) framework, which describes a global trade-off between “conservative’’ and “acquisitive’’ leaf traits (Donovan et al. 2011; Reich 2014; Shipley et al. 2006; Wright et al. 2004). Conservative leaves are typically thick, dense, and have a low specific leaf area (SLA). They are long‑lived, stress‑tolerant, with low photosynthetic rates, low leaf nitrogen (N, reflecting RUBISCO content), and high water‑use efficiency. In contrast, acquisitive leaves are typically thin with a high SLA. They are metabolically active, have high leaf N, and are structured to maximize rapid resource capture, but they require a reliable resource supply. Epiphytes are generally at the conservative end of the LES due to limited water and nutrient availability, while terrestrial plants are more to the acquisitive end of the LES, as they tend to have more stable and higher supplies of water and nutrients (Benzing 1990; Helsen et al. 2023).

Empirical studies largely confirm the LES differences between epiphytes and terrestrial plants. Epiphytes tend to exhibit thick leaves, reduced stomatal density, lower stomatal conductance, and higher water-use efficiency than non-epiphytic plants, because they guard against desiccation and nutrient scarcity to a greater degree (Campany et al. 2021; Gotsch et al. 2015; Holbrook and Putz 1996). Terrestrial plants, in contrast, usually have higher SLA, greater stomatal conductance, and higher photosynthetic rates, reflecting acquisitive strategies that enhance carbon gain when water and nutrients are reliable (Zhang et al. 2023). Comparative analyses across life forms further illustrate that epiphytes often have more-enriched foliar *δ*^13^C values, indicating tighter stomatal regulation associated with more limited water supply, whereas terrestrial plants maintain higher photosynthetic capacity and more rapid growth under favorable conditions (Hietz et al. 2022; Watt and Watkins 2021).

However, most of these insights on the differences in LES between epiphytes and terrestrial plants are derived from interspecific comparisons, which can confound environmental effects with phylogenetic differences. The transition between these habitats is fundamentally limited by the trade-off between the high resource-gathering efficiency needed for the terrestrial environment and the extreme water-conservation traits required for the “functional desert” of the canopy (Hietz et al. 2022; Zotz and Hietz 2001). Furthermore, the high cost of maintaining the phenotypic plasticity necessary to handle disparate light levels, nutrient availabilities, and mechanical requirements likely leads to specialization for a single environment (Hu et al. 2025; Zotz 2016). One noticeable exception is a study on the intraspecific and interspecific functional trait differences, foliar nitrogen content, *δ*^15^N, and *δ*^13^C in four fern species growing across epiphytic, epipetric, and terrestrial habitats (Watts and Watkins 2021). The results of this study provide partial support for the divergence in LES between epiphytes and terrestrial plants and highlight the importance of quantifying including intraspecific trait variation in studies that assess trait variation of plants growing in diverse habitats. Part of the scarcity of comparing trait divergence between epiphytic and terrestrial individuals of the same species is because there are few species that can grow in both epiphytic and terrestrial habitats. For example, a survey of 264 ferns along an elevation gradient in Costa Rica found that only one species could grow in both habitats (Watkins et al. 2006). Trait differences observed between species along environmental gradients may reflect either plastic responses to current habitat conditions or differences arising from their distinct evolutionary histories (phylogeny). Thus, the scarcity of conspecific comparisons across terrestrial and epiphytic individuals limits our understanding of the extent to which LES-related differences arise from plasticity versus evolutionary divergence.

*Nephrolepis cordifolia* (L.) C. Presl, a widespread tropical fern in the Polypodiales, is ideal for addressing conspecific divergence in leaf economical traits between epiphytic and terrestrial individuals. This widespread fern can grow both epiphytically on tree trunks and branches and terrestrially in soil. Moreover, epiphytic and terrestrial individuals often grow in proximity, with similar broad scale environmental conditions (e.g., temperature, humidity, precipitation) and thus provide a natural experimental setting for examining how individuals of the same species adjust leaf structure, gas exchange rates, and water-use strategies in the contrasting epiphytic and terrestrial microhabitats. Because phylogeny and broad environmental conditions are consistent, the divergence in morphological and physiological response can be more directly attributed to epiphytic and terrestrial conditions.

Several traits are particularly informative for identifying position along the LES because they reflect morphological and physiological adjustments to contrasting microhabitats. Leaf thickness, SLA, and leaf dry matter content (LDMC) reflect leaf structural investment strategy and the carbon economies of leaf tissue construction, functionally distinguishing conservative from acquisitive strategies (Poorter 2002, 2009; Ren et al. 2022). Stomatal density and size regulate the trade-off of carbon assimilation and water loss, affecting photosynthetic capacity and water-use efficiency (Bertolino et al. 2019; Sekiya and Yano 2008). The quantum yield of photosystem II (Φ_*PSII*_), reflects photosynthetic performance under variable light regimes (Skillman 2008; Sofo et al. 2009). The maximum quantum yield of PSII, *F*_v_/*F*_m_, is strongly correlated with photosynthetic performance (Baker 2008), and is widely used as an indicator of plant stress responses (Abbas et al. 2019; Barboričová et al. 2022; Parkhill et al. 2001). Foliar *δ*^13^C provides an integrated measure of long-term water-use efficiency, plant status (i.e., drought stress), and stomatal behavior in response to the environment (Farquhar et al. 1989; Simioni 2023). In addition to these structural and gas-exchange traits, leaf chlorophyll a and b and nutrient (C, N, P, K, Ca, Mg) concentrations offer insight into how plants allocate resources to optimize photosynthesis, e.g., light harvesting and biochemical function (Evans 1989; Tränkner et al. 2018). Together, these traits form a multidimensional assessment of how *N. cordifolia* responds to contrasting epiphytic and terrestrial microhabitats by adjusting leaf carbon assimilation, water use, and structural investment.

Given the soil and air microclimate differences between epiphytic and terrestrial habitats, and the trait-based framework of LES theory, this study evaluates how individuals of *N. cordifolia* adjust their leaf physiology and morphology across these two microhabitats. We tested five hypotheses. Due to a more limited and intermittent water supply in canopy habitats, we hypothesized that epiphytic individuals would show higher water-use efficiency (*H*_1_) and possess lower stomatal density (*H*_2_) and smaller stomatal size (*H*_3_) than terrestrial individuals. Additionally, due to the lack of direct access to soil nutrients, epiphytic individuals should have lower foliar nutrient concentrations than terrestrial individuals (*H*_4_). Lastly, because of water and nutrient constrains, we hypothesized that epiphytic individuals would exhibit lower quantum efficiency than terrestrial individuals (*H*_5_). Our hypotheses were motivated by two different rationales: some (*H*_1_–*H*_3_) were derived from expectations of the evolved LES syndrome in epiphytes, while others (*H*_4_–*H*_5_) were based on ecological consequences of resource limitation in canopy habitats. However, because our study is based on field comparisons, the results cannot distinguish whether observed differences reflect evolved strategies or plastic responses to environmental constraints. Thus, the classification of hypotheses reflects their conceptual basis rather than an empirical separation. Nonetheless, our study has broad implications for fern physiological functioning with climate change, as shifts toward increased drought stress and greater irradiance levels in most tropical wet forests will differentially challenge canopy-dwelling and terrestrially rooted plant populations (Zotz 2016). In addition, by integrating morphological, physiological, and isotopic measurements, this study contributes to a more comprehensive understanding of how environmental conditions shape intraspecific leaf variation within the LES.

## Materials and methods

### Study species

**Fig. 1:**
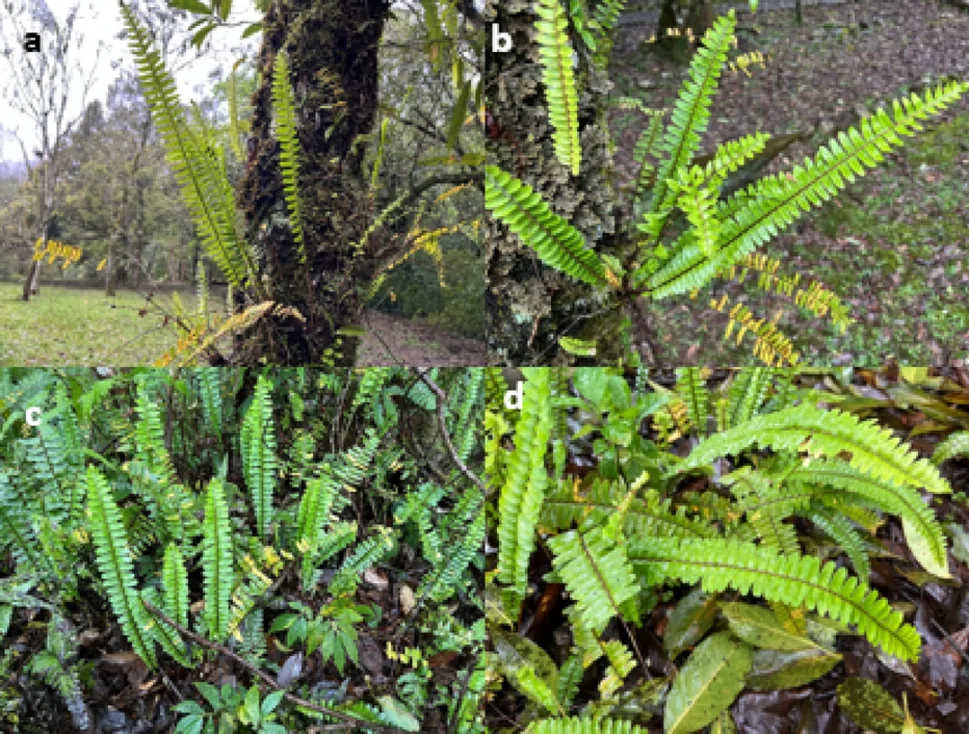
Epiphytic (**a**), (**b**) and terrestrial (**c**), (**d**) individuals of *Nephrolepis cordifolia*

*N. cordifolia* (L.) Presl (Nephrolepidaceae) is a facultative epiphyte (Fig. 1), a term commonly applied to species that can grow both epiphytically and terrestrially, in contrast to obligate epiphytes that are restricted to host trees throughout their life cycle. According to The Flora of Taiwan, it is a widespread fern with tufted stipes 5–15 cm long, often producing shiny stolons and scaly tubers (Shieh et al. 1994). Its fronds, 30–60 cm in length, are pinnate with sessile pinnae that vary at the base (can be auriculate, finger-like, or triangular in shape), serrulate to crenate margins, and sori that are round with reniform indusia. The rachis is grooved and scaly above, while the pinnae are articulate to the rachis and become gradually shortened toward the base. *N. cordifolia* can grow on tree trunks (epiphytic), on the ground (terrestrial), and occasionally on fallen logs or rocks. In this study, we compared epiphytic and terrestrial individuals.

### Study site

**Fig. 2:**
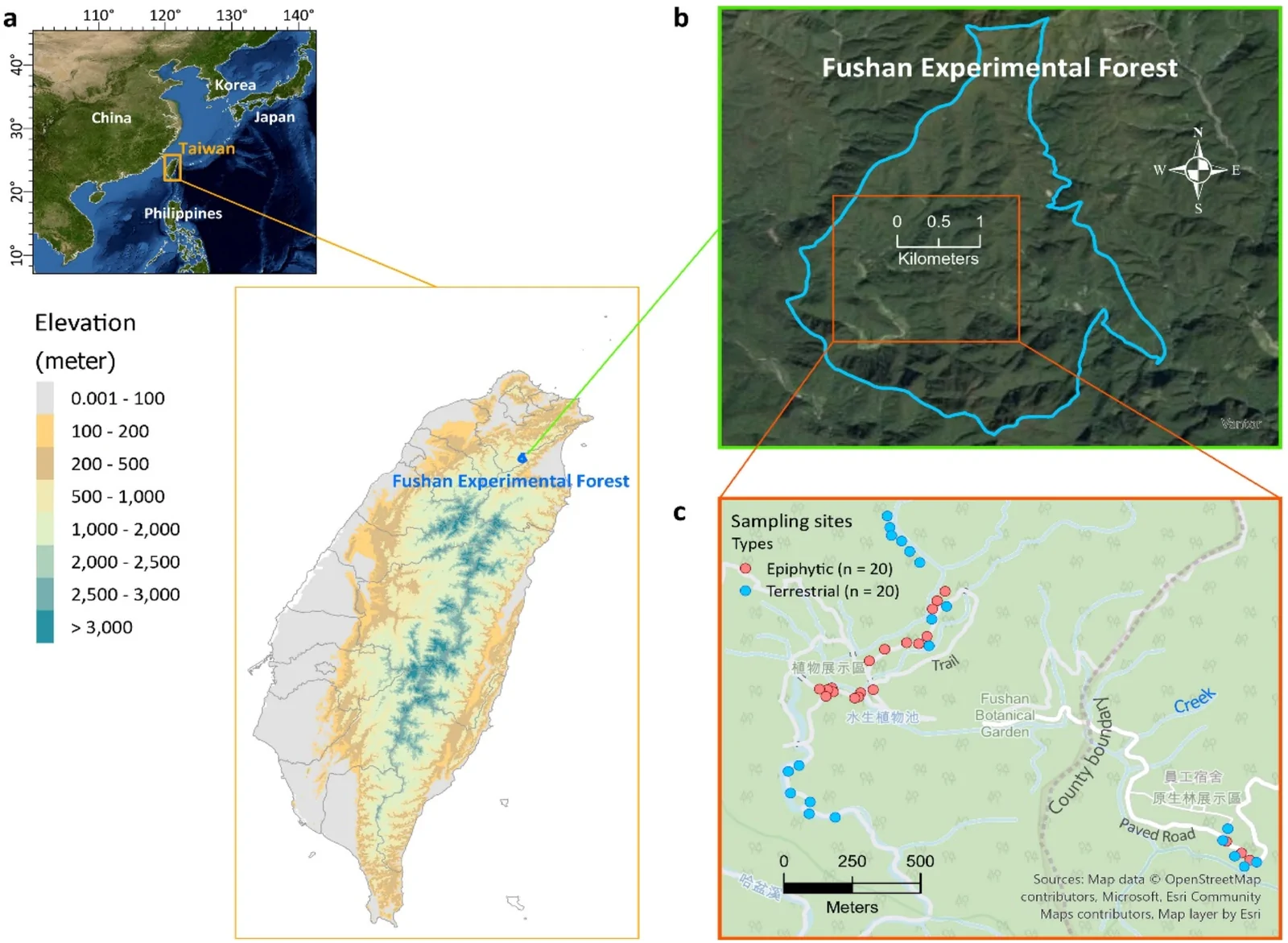
Distribution of studied plants at the Fushan Experimental Forest, northeastern Taiwan

Plants used for this study were located at the Fushan Experimental Forest (FEF) of northeastern Taiwan (Fig. 2). The FEF is a low-elevation (700^−1^,400 m) subtropical rainforest, with an annual mean temperature of 18.0 °C (11.5 °C in January and 23.7 °C in July), mean annual precipitation of 4,037 mm (2,540–7,400) and monthly mean relative humidity greater than 90% throughout the year (from 1994 to 2022; Chang et al. 2024). The forest has a multi-layer structure dominated by Lauraceae and Fagaceae tree species. A total of 515 species, belonging to 329 genera and 124 families of vascular plants are recorded in the FEF (TFRI 1989). Among which, 65 are epiphyte species belonging to 20 families and 42 genera, representing 12% of the recorded vascular plant species in the FEF.

*N. cordifolia* is abundant at the FEF. While *N. cordifolia* prefers open environment, some terrestrial and epiphytic individual can also be found in the forest interior. The relative abundance of epiphytic and terrestrial individuals exhibited high spatial heterogeneity across the study area, with no consistent landscape-level dominance by either growth form. In many instances, we observed a continuous distribution where terrestrial colonies extended from the surrounding understory to the immediate base of the tree, while epiphytic individuals occupied the trunk from the root collar to several meters above the ground. This spatial overlap and the lack of a clear habitat bias underscore the truly facultative nature of *N. cordifolia* in this forest.

### Plant sampling

Twenty epiphytic and 20 terrestrial individuals of *N. cordifolia* were selected for this study. The plants distributed in two locations, 1.5 km apart, at the FEF (Fig. 2). All studied individuals were selected from forest edges. Insolation, relative humidity and daily temperatures are environmental factors that play important roles in characterizing plant traits. Direct and indirect (diffused) light availabilities, which are closely related to Insolation, relative humidity and temperatures, of the studied plants were estimated using hemispherical photography. One hemispherical photograph was taken directly at the center of each studied plant and analyzed using HemiView (Delta-T 2000). Direct and indirect light availability is the relative (proportional) direct and light available at the point of measurement relative to that above tree canopy (Rich 1990).

Each plant was collected from a host tree for epiphytic individuals or from the ground for terrestrial individuals, with a minimum spacing of 10 m between sampled plants. Epiphytic individuals were located at least 1.5 m above ground and confirmed to have no root connection to soil. A newly matured, undamaged frond (40–50 cm in length) without sori was collected from each plant for measurements of frond morphology, carbon isotopes, nutrient concentrations and chlorophyll concentrations, while photochemical efficiency was measured in the field.

### Frond morphology

We measured specific leaf area (SLA, mm^2^ mg⁻^1^), leaf dry matter content (LDMC, mg g⁻^1^), stomatal traits, and frond thickness. Five pinnae were removed from each frond and weighed for fresh mass using a precision balance (Shimadz, Taipei, Taiwan). Leaf area was determined from scanned images using ImageJ (Rueden et al. 2017). Samples were dried in an oven at 45 °C to constant mass to obtain dry weight. SLA was calculated as leaf area divided by dry mass, and LDMC as dry mass divided by fresh mass. Stomatal density was measured from clear nail polish impressions of the abaxial surface mounted on microscope slides. Number of stomata were counted from four randomly selected fields of view (avoiding the midrib and margins), under a compound microscope at 100 × magnification. Stomatal densities (no. mm^−2^) of the four fields were averaged. Stomatal length and width were measured at 400 × magnification using images from three randomly selected fields of view of each pinna and measured with ImageJ (units: μm^2^). The stomatal dimension measurements included the guard cells and were taken at night when the stomata are closed (or at minimum aperture) to minimize the noise of fluctuating light or humidity levels. Frond thickness was measured from one pinna each at the basal, middle, and apical portions of a newly matured frond using a thickness gauge (Mitutoyo 7313; Kawasaki, Japan, precision 0.01 mm).

### Frond carbon isotopes and nutrient concentrations

Dried fronds were ground into fine powder (particle size less than 0.5 mm). Finely ground frond samples (4.5–5 mg) were enclosed in tin capsules and analyzed using an isotope ratio mass spectrometer (IRMS) coupled to an ANCA-GSL elemental analyzer (PDZ Europa Ltd., Cheshire, UK) located at the University of California-Davis. Carbon isotope ratios were calculated from the ratio in the sample and the standard reference as:

$$ \delta^{{{13}}} {\mathrm{C}} = ({\mathrm{R}}_{{{\mathrm{sample}}}} /{\mathrm{R}}_{{{\mathrm{standard}}}} - {1}) \, \times {1}000 $$

where the standard reference ratio corresponds to Pee Dee Belemnite (Farquhar et al. 1989).

Approximately 4 mg of dried frond material was used for C and N measurement using a CHNOS Elemental Analyzer (Elementar Americas Inc., Ronkonkoma, NY USA). For P, K, Ca, and Mg analysis, 0.5 g of sample powder was digested and quantified using inductively coupled plasma atomic emission spectroscopy (JY 2000, Horiba Scientific, Longjumeau, France).

### Chlorophyll concentration and photochemical efficiency

Subsamples of the selected fronds were scanned for leaf area determination, then homogenized in 95% ethanol. Extracts were transferred into 15 mL centrifuge tubes, sealed with Parafilm, wrapped in aluminum foil, and stored at 4 °C in darkness for 24 h. After recording solution volume, samples were centrifuged at 4,000 rpm for 10 min (Allegra X-12R, Beckman Coulter, Brea, CA USA). Supernatant absorbance was measured at 430, 649, and 665 nm using a UV/VIS spectrophotometer (Du 640, Beckman Coulter, Brea, CA USA). Absorbance at 430 nm was maintained between 0.7–0.9 by dilution or reprocessing, if necessary. Chlorophyll a, chlorophyll b, total chlorophyll, and chlorophyll *a:b* ratios per unit area and per unit dry mass were calculated following Wintermans and De Mots (1965).

The maximum quantum yield of PSII (*F*_v_/*F*_m_) was measured using a MINI-PAM Photosynthesis Yield Analyzer (ICT International, New South Wales, Australia). Prior to measurement, each frond was dark-adapted for at least 30 min by covering it with aluminum foil. Fluorescence was then recorded from a pinna located at the mid-section of the frond. The maximum quantum yield was calculated as *F*_v_/*F*_m_ = (*F*_m_ − *F*_0_)/*F*_m_, where *F*_0_ represents the background fluorescence of the dark-adapted frond and *F*_m_ is the maximum fluorescence yield obtained when a saturating light pulse of 10,000 μmol m^−2^ s^−1^ was applied for 0.8 s.

### Data analysis

Data normality of each variable was examined using the Shapiro–Wilk tests. All morphological and physiological measurements were first tested for differences between epiphytic and terrestrial individuals using two-sample *t*-tests. When the data did not meet the assumption of normal distribution, the Mann–Whitney U test was used instead, with statistical significance set at *α* = 0.05. All statistical analyses were conducted using R v.3.6.1 (R Core Team 2019).

## Results

### Light availability

No significant differences were found between epiphytic and terrestrial individuals in indirect light availability (epiphytic: 0.272 ± 0.023 (mean ± error); terrestrial: 0.214 ± 0.026; *t* = 1.697, *p* = 0.098) or direct light availability (epiphytic: 0.311 ± 0.032; terrestrial: 0.246 ± 0.031; *t* = 1.438, *p* = 0.159).

### Frond morphology

**Fig. 3:**
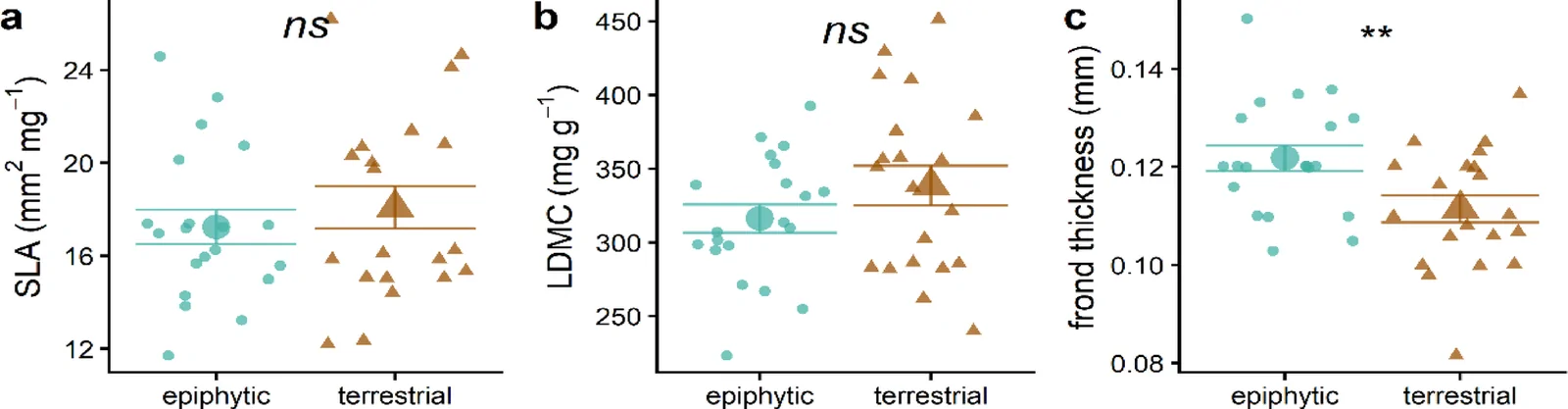
Leaf morphological traits, **a** specific leaf area (SLA), **b** leaf dry matter content (LDMC), and **c** frond thickness, of epiphytic and terrestrial individuals of *N. cordifolia*. SLA: specific leaf area; LDMC: leaf dry matter content; NS: not significant and ** indicates significant net difference at *α* = 0.01. The large symbols and error bars are means ± standard error

**Fig. 4:**
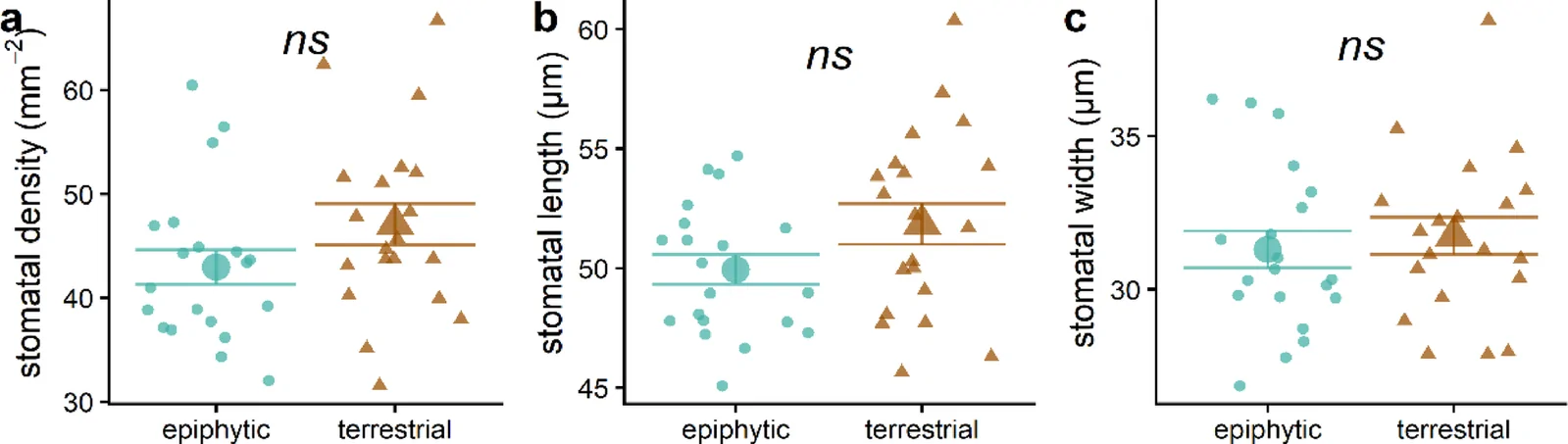
Stomatal density and stomatal dimensions (length and width) in epiphytic and terrestrial *N. cordifolia*. NS: not significant. The large symbols and error bars are means ± standard error

SLA and LDMC did not differ significantly between epiphytic and terrestrial individuals of *N. cordifolia* (Fig. 3a, b). In contrast, fronds were thicker in epiphytic individuals than terrestrial individuals (Fig. 3c). Stomatal density and dimensions (length and width) showed no significant differences between the epiphytic and terrestrial individuals (Fig. 4).

### ***δ***^***13***^***C and frond nutrient concentrations***

Leaf stable carbon isotopic composition (*δ*^13^C) was significantly greater in epiphytic individuals than in terrestrial individuals (Table 1). Foliar carbon concentrations were significantly higher in epiphytic individuals compared to terrestrial individuals (Table 1). In contrast, terrestrial individuals exhibited higher concentrations of nitrogen and magnesium (Table 1). Therefore, C:N ratios differed significantly between habitats, with epiphytic individuals exhibiting higher values than terrestrial individuals (Table 1). Concentrations of other elements did not differ significantly between epiphytic and terrestrial individuals, and N:P ratios were statistically similar (Table 1).

**Table 1:** Foliar element concentration and ratios and *δ*^13^C (mean ± standard error) of epiphytic and terrestrial individuals of *Nephrolepis cordifolia*

Element	Epiphytic	Terrestrial	*p* value
Ca (mg g^−1^)	6.93 ± 0.49	7.74 ± 0.71	0.3531
**Mg (mg g**^**−1**^**)**	**4.25 ± 0.22**	**6.10 ± 0.33**	**< 0.001**
K (mg g^−1^)	28.84 ± 1.25	31.24 ± 1.49	0.2258
P (mg g^−1^)	2.10 ± 0.21	2.20 ± 0.14	0.3689
**C (mg g**^**−1**^**)**	**428.14 ± 1.27**	**419.24 ± 4.35**	**0.0042**
**N (mg g**^**−1**^**)**	**15.17 ± 0.63**	**17.91 ± 0.77**	**0.009**
**C:N**	**29.07 ± 1.09**	**24.21 ± 1.05**	**0.0026**
N:P	8.13 ± 0.59	8.59 ± 0.47	0.5408
***δ***^***13***^**C**	**− 29.23 ± 0.19**	**− 30.83 ± 0.13**	**< 0.001**

Elements with statistically different concentrations between habitats *(p*-values < 0.05*)* are marked in bold

### Chlorophyll concentration and photochemical efficiency

**Fig. 5:**
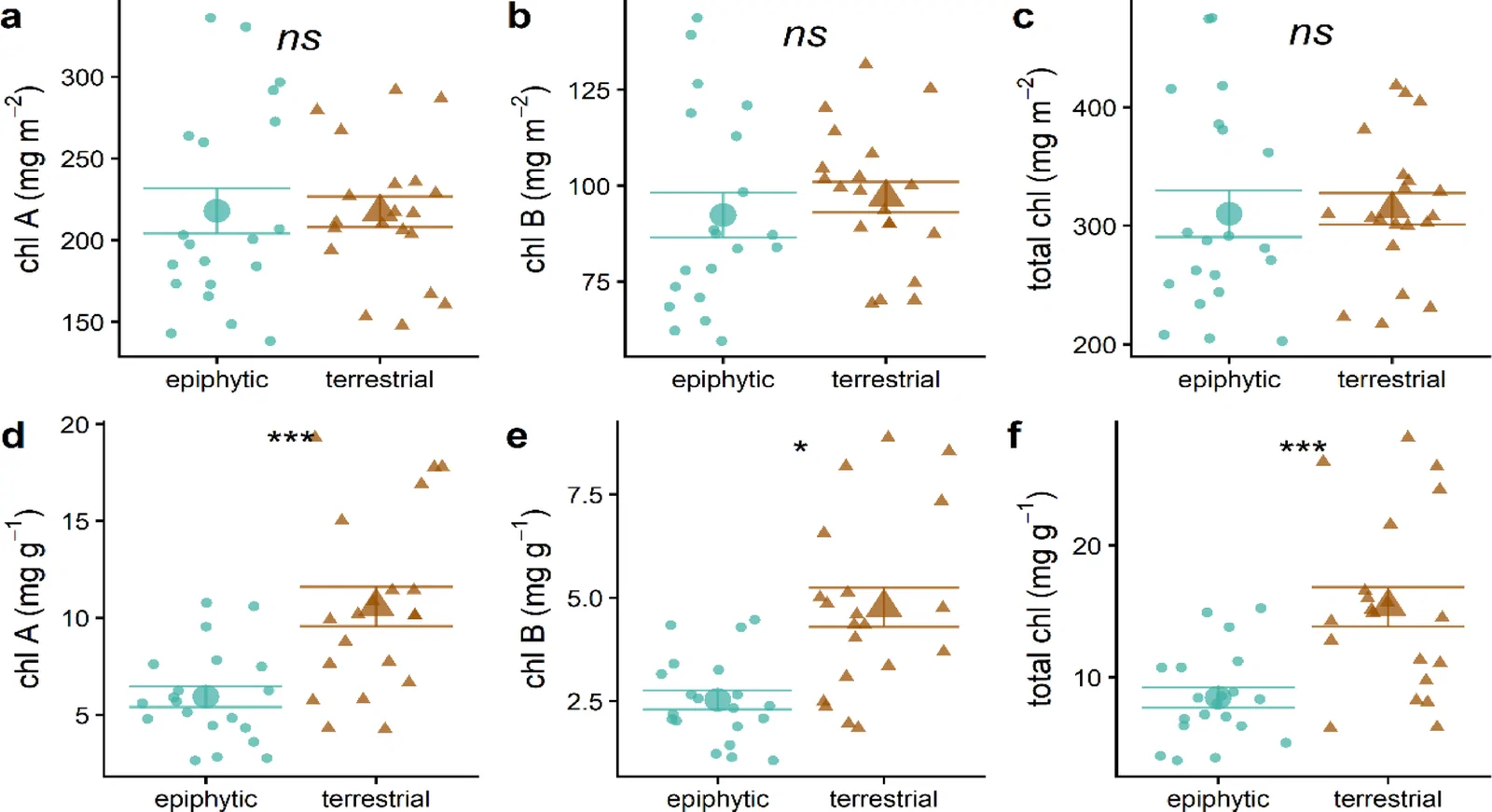
Chlorophyll concentrations per unit area (**a**), (**b**), (**c**) and per unit dry mass (**d**), (**e**), (**f**) in epiphytic and terrestrial *N. cordifolia*. NS: not significant and *, and *** indicate significant net difference at α = 0.05, and 0.001, respectively. The large symbols and error bars are means ± standard error

**Fig. 6:**
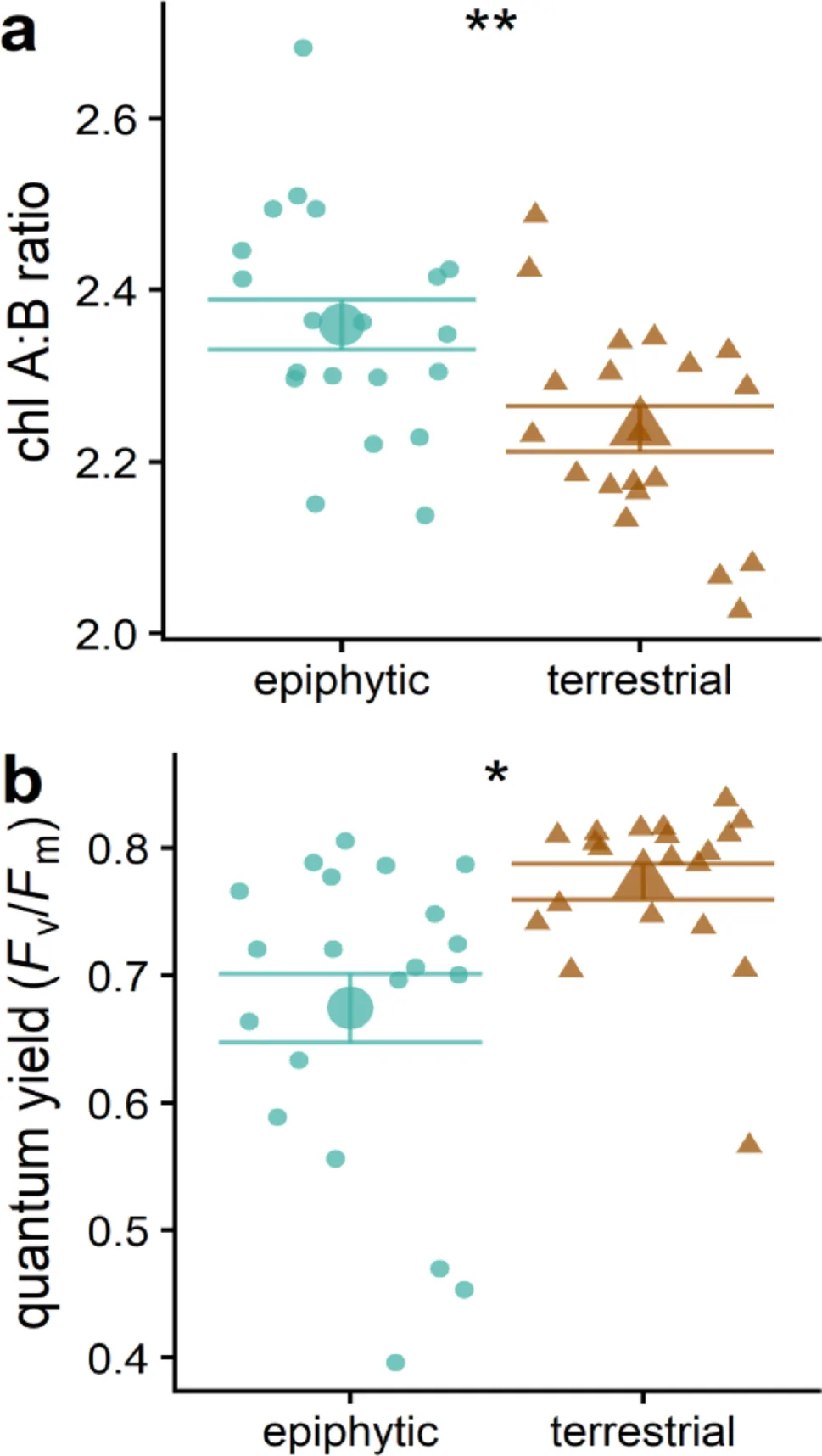
Chlorophyll *a:b* ratio, and maximum quantum yield (*F*_v_/*F*_m_) of epiphytic and terrestrial individuals of *N. cordifolia.* *, ** indicate significant net difference at α = 0.05 and 0.01, respectively. The large symbols and error bars are means ± standard error

Area-based chlorophyll a, chlorophyll b, and total chlorophyll concentrations were similar between epiphytic and terrestrial individuals (Fig. 5a–c). However, on a mass basis, terrestrial individuals exhibited substantially higher concentrations of chlorophyll a, chlorophyll b, and total chlorophyll than epiphytic individuals (Fig. 5d–f), while the chlorophyll *a:b* ratio was significantly higher in epiphytic individuals than in terrestrial individuals (Fig. 6a). *F*_v_/*F*_m_ was also significantly higher in terrestrial plants than in epiphytic plants, but not all epiphytic individuals were uniformly low, with three individuals having extremely low values (less than 0.5, Fig. 6b).

## Discussion

Although epiphytic habitats are often characterized by higher light exposure compared to the forest floor, our results showed no significant differences in either indirect or direct light availability between the two groups. This suggests that the sampled individuals, which were all located near forest edges, experienced a relatively homogenous light environment regardless of their growth substrate.

### Frond morphology and structural traits

Frond morphologies were broadly similar between terrestrial and epiphytic individuals, with SLA, LDMC, and stomatal density not differing significantly. The lack of significant difference in SLA between epiphytic and terrestrial individuals (Fig. 3) may be partially explained by the similarity in light availability across habitats. Typically, lower SLA is associated with high-light environments to prevent photoinhibition; however, because direct and indirect light availability did not differ significantly between growth habitats in this study, the observed increases in frond thickness among epiphytes are more likely due to differences in water and nutrient availability rather than a response to light intensity. Our result contrasts with global LES patterns that place epiphytes on the conservative end of the spectrum with lower SLA and higher LDMC (Gotsch et al. 2015; Guzmán‐Jacob et al. 2022; Richards and Damschen 2022). The lack of differentiation in these traits suggests that *N. cordifolia* maintains a relatively stable level of structural investment across habitats, possibly reflecting genetic or architectural constraints (Bateman 1999; Niklas 1986). In contrast, leaf thickness exhibited clear differentiation, with epiphytic individuals producing thicker fronds, consistent with expectations for plants exposed to more variable and drier canopy microclimates (Wright et al. 2004). Increased thickness can enhance water storage, slow dehydration, and reinforce tissues under rapid fluctuations in humidity (Mauseth 2000; Nguyen et al. 2017). The fact that thickness differs, whereas SLA and LDMC do not, suggests that *N. cordifolia* may adjust specific anatomical components, such as mesophyll volume or cuticle development, while maintaining similar overall construction costs. While common garden or reciprocal transplant experiments would be essential to distinguish plastic responses from fixed adaptations, such approaches are difficult for *N. cordifolia*, as transplanting individuals between canopy and terrestrial habitats is technically challenging and associated with high mortality.

Unlike studies reporting low stomatal density in vascular epiphytes as an adaptation to reduce water loss (Dunn et al. 2019; Li et al. 2020; Watkins et al. 2010), we found no differences in stomatal density or dimension between habitats. This suggests that *N. cordifolia* does not rely heavily on stomatal structural adjustments to respond to canopy conditions, possibly because stomatal traits are less plastic than other physiological responses such as photosynthesis and respiration (Qu et al. 2016; Wang et al. 2019). The lack of divergence in stomatal morphology indicates that the species may instead adjust physiology rather than anatomy when acclimating to canopy versus terrestrial conditions.

### Chlorophyll content and photochemical performance

Epiphytic individuals did not exhibit higher photosynthetic efficiency, instead, terrestrial individuals had greater *F*_v_/*F*_m_ values, indicating less photo-inhibition and higher potential quantum yield. This is consistent with findings that terrestrial habitats impose less stress on photosystems compared to canopy habitats (Ivanova et al. 2024; Zhang et al. 2009). Mass-based chlorophyll concentrations were also higher in terrestrial individuals, supported by their greater nitrogen and magnesium availability, both essential for chlorophyll synthesis and photosynthetic enzyme function (Ciećko et al. 2012). This pattern is further explained by the structural trade-off between frond thickness and tissue density. Because SLA remained stable despite thicker epiphytic fronds, the epiphytes mathematically possess a lower internal tissue density. This lower density implies an expansion of non-chlorophyllous structural or vacuolated spaces in epiphytes, which dilutes pigments on a mass basis and explains the higher mass-based concentrations found in the denser terrestrial fronds. Interestingly, area-based chlorophyll concentrations did not differ, suggesting that epiphytes maintain comparable pigment density per surface area but distribute pigments across thicker tissues.

Although lower chlorophyll *a:b* ratios are often interpreted as an adaptation to shaded understory conditions (Hoober and Argyroudi-Akoyunoglou 2004; Kume et al. 2018), our analysis of hemispherical photography showed no significant differences in direct or indirect light availability between the two habitats. This suggests that the lower chlorophyll *a:b* ratio and higher mass-based chlorophyll concentrations in terrestrial individuals are unlikely a plastic response to light intensity. Instead, these differences are consistent with the superior nutrient status of terrestrial individuals, which have direct access to soil resources. Enhanced nitrogen and magnesium availability in the terrestrial habitat allow for greater investment in light-harvesting complexes, improving light-use efficiency even when the ambient light environment is similar to that of the epiphytic habitat (Evans 1989; Hoober et al. 2007).

### Nutrient concentrations and stoichiometric patterns

Clear differences in foliar nutrient concentrations were observed between habitats. Terrestrial individuals had higher nitrogen and magnesium concentrations, while epiphytes exhibited higher carbon content and C:N ratios. These differences may reflect contrasting nutrient acquisition strategies. Terrestrial individuals benefit from direct soil access, enabling acquisitive investment in pigments and enzymes, whereas epiphytes rely on intercepted debris and atmospheric inputs (Zotz 2016). Alternatively, the differences in nutrient concentrations may be due to higher nutrient availability of terrestrial individuals as they can, while epiphytic individuals cannot, access nutrients in the soil. Nonetheless, higher C:N ratios in epiphytes indicate greater investment in carbon-rich structural compounds and slower nutrient turnover, consistent with conservative leaf construction typical of nutrient-limited environments (Grasset et al. 2015).

### Water-use efficiency and stomatal regulation

The *δ*^13^C results indicate substantially higher intrinsic water-use efficiency in epiphytic individuals and tighter stomatal regulation. Although the FEF is characterized by consistently high relative humidity (Lin et al. 2024), previous studies have shown that epiphytic plants can still experience intermittent water limitation due to the absence of direct soil water access and the occurrence of short rainless periods lasting several days (Huang and Lin 2016; Martin 2010). Such episodic drying cycles may impose stronger water stress on epiphytic individuals than on terrestrial plants, despite similar ambient humidity. Microclimatic temperature differences between epiphytic and terrestrial positions are likely minor relative to differences in water availability and thus unlikely to be a primary driver of the observed trait variation.

Notably, stomatal density and size did not differ between habitats, indicating that long-term water-use divergence was driven by stomatal behavior, rather than anatomy. Because *δ*^13^C integrates stomatal behavior over the lifespan of a leaf (Brooks et al. 1997; Körner et al. 1988), its relative enrichment in epiphytic ferns supports sustained physiological adjustment to greater evapotranspirative demand in canopy than terrestrial microenvironments (Pypker et al. 2017). In contrast, terrestrial individuals experience greater water availability, leading to lower *δ*^13^C values characteristic of faster carbon assimilation under less constrained stomatal regulation.

### Integrating across the LES framework

Collectively, the observed trait differences are consistent with key LES contrast between conservative and acquisitive strategies. The significantly higher *δ*^13^C values in epiphytic individuals indicate higher water-use efficiency in canopy habitats. Although stomatal density and dimensions did not differ, higher *δ*^13^C confirms that epiphytes regulate stomata more conservatively across their lifespan, highlighting physiological plasticity without structural change.

Taken together, thicker leaves, higher C:N ratios, higher *δ*^13^C, and reduced *F*_v_/*F*_m_ point toward a more conservative ecophysiological profile in epiphytic individuals. In contrast, terrestrial individuals exhibit more acquisitive traits—higher chlorophyll concentrations, greater nutrient availability, higher photochemical efficiency, and lower intrinsic water-use efficiency. These differences reflect divergent microenvironmental constraints and highlight the capacity of *N. cordifolia* to occupy both ends of the LES through phenotypic and physiological plasticity.

### Hypotheses revisited

Taken together, our results provide differentiated support for our original hypotheses. We found strong evidence that epiphytic individuals exhibited higher water-use efficiency (*H*_1_), as indicated by enriched *δ*^13^C values, which supported sustained stomatal regulation under greater evaporative demand in the forest canopy. In contrast, we found no evidence that epiphytic individuals possess lower stomatal density (*H*_2_) or smaller stomatal size (*H*_3_) than terrestrial individuals, rejecting expectations of structural stomatal adjustment. Differences in frond nutrient concentrations and stoichiometry were consistent with contrasting acquisition strategies, supporting that epiphytic individuals likely experienced nutrient limitation compared to terrestrial individuals (*H*_4_). Lastly, terrestrial individuals exhibited significantly higher *F*_v_/*F*_m_ values, indicating greater photochemical efficiency and reduced stress compared to epiphytic individuals, which supported that epiphytic individuals operated under lower quantum efficiency (*H*_5_). Overall, our study provides support for water-use and nutrient-related predictions, whereas stomatal structural hypotheses were not supported, underscoring the dominance of physiological over anatomical regulation of gas exchange and highlighting the context-dependent nature of LES expression within species. These outcomes highlight the importance of physiological plasticity in *N. cordifolia*. However, because our study is based on field comparisons, the observed differences cannot be definitively attributed to evolved strategies versus environmental constraints. Future common garden or reciprocal transplant experiments would be necessary to disentangle these mechanisms.

### Implications under global climate change

The enriched *δ*^13^C values together with reduced *F*_v_/*F*_m_ in epiphytic individuals suggest that epiphytic individuals may operate closer to their physiological constraints, potentially leaving less buffering capacity under future drought and irradiance. Projected increases in drought, vapor pressure deficit, and irradiance will likely exacerbate these stresses, reducing epiphytic plant performance and survival. Nutrient limitation in canopy substrates may further constrain chlorophyll synthesis and photosynthetic function. Terrestrial individuals, with higher nutrient availability and lower *δ*^13^C values, may be more resilient to climate extremes. As a result, facultative epiphytes such as *N. cordifolia* may shift toward terrestrial habitats under future climate scenarios. Overall, these results underscore the importance of microhabitat-specific strategies in shaping plant responses to environmental change. By revealing intraspecific differences in plant form and function across habitats, this study highlights how physiological plasticity, trait trade-offs, and environmental stress interact to determine resilience of canopy and terrestrial populations.

## Data Availability

The data supporting the findings are available at depositar repository. https://pid.depositar.io/ark:37281/k5w1j9g3w

## Abbreviations

PSII: Photosystem II
*δ*^13^C: Carbon stable isotope ratio
LDMC: Leaf dry matter content
LES: Leaf economic spectrum
SLA: Specific leaf area
